# Reasons why nurses decline influenza vaccination: a qualitative study

**DOI:** 10.1186/s12912-017-0215-5

**Published:** 2017-04-28

**Authors:** Anina Pless, Stuart R. McLennan, Dunja Nicca, David M. Shaw, Bernice S. Elger

**Affiliations:** 1 Institute for Biomedical Ethics, University of Basel, Bernoullistrasse 28, 4056 Basel, Switzerland; 2 Institute for History, Ethics and Philosophy of Medicine, Medizinische Hochschule Hannover, Carl-Neuberg-Str. 1, 30625 Hannover, Germany; 3 Institute of Nursing Science, University of Basel, Bernoullistrasse 28, 4056 Basel, Switzerland

**Keywords:** Attitudes, Influenza, Nurses, Qualitative research, Vaccination

## Abstract

**Background:**

To explore reasons of non-vaccinated nursing staff for declining seasonal influenza vaccination. The annual influenza vaccination of healthcare workers reduces morbidity and mortality among vulnerable patients. Still, vaccination rates remain very low, particularly in nursing staff. While several studies have explored barriers for healthcare workers to get vaccinated, most have used a quantitative approach.

**Methods:**

Data were collected by in-depth individual semi-structured interviews with 18 nurses from a range of fields, positions in organizational hierarchy, work experience and hospitals in two German-speaking cantons in Switzerland. Interviews were transcribed and analysed using conventional content analysis.

**Results:**

Three interconnected themes explaining why nurses decline influenza vaccination were identified: Firstly, the idea of maintaining a strong and healthy body, which was a central motif for rejecting the vaccine. Secondly, the wish to maintain decisional autonomy - especially over one's body and health. Thirdly, nurses' perception of being surrounded by an untrustworthy environment, which restricts their autonomy and seemingly is in opposition to their goal of maintaining a strong and healthy body.

**Conclusion:**

Nurses tend to rely on conventional health beliefs rather than evidence based medicine when making their decision to decline influenza vaccination. Interventions to increase influenza vaccination should be tailored specifically for nurses. Empowering nurses by promoting decision-making skills and by strengthening their appraisal may be important factors to consider when planning future interventions to improve vaccination rates. The teaching of evidence-based decision-making should be integrated on different levels, including nurses' training curricula, their workspace and further education.

## Background

Despite explicit recommendations by public health authorities and studies stating that annual influenza vaccination of healthcare workers (HCWs) is associated with a reduction of morbidity and mortality among patients [1], vaccination rates among HCWs worldwide are low, with only about 4–40% coverage rates being achieved [2–4]. Interestingly, rates among nurses - the HCWs in closest contact with patients - are remarkably lower than those of physicians [5–7]. Vaccination rates in Switzerland are similarly low, with studies finding about 15% coverage rates among nurses [8, 9].

Multiple studies have been conducted worldwide to examine the reasons why HWCs decline the influenza vaccination. Reasons include concerns about adverse reactions, perceived lack of susceptibility, and alleged lack of vaccine effectiveness [4, 5, 10–14]. While there have been several studies internationally exploring barriers for HCWs to get vaccinated, to our knowledge, most studies have used a quantitative survey approach [14, 15]. The few qualitative studies were conducted in the USA [10, 16].

Studies show that educational interventions as well as providing easier access to the influenza vaccine raise vaccination rates among physicians but have very little effect on nursing staff [7, 8]. For the development of effective interventions it is especially important to better understand nurses’ perception and to identify specific barriers in this group of healthcare workers. This survey therefore aims to explore reasons for declining the influenza vaccination in nursing staff via qualitative interviews. By letting nursing staff discuss their experiences and factors preventing them from getting vaccinated, we expect to obtain more in-depth information on this subject. This kind of evidence generation is recommended for the development of complex behavioural change interventions [17].

## Methods

### Setting and recruitment procedures

Non-vaccinated participants were recruited from several nursing departments in two teaching hospitals in the German speaking part of Switzerland. The administrators of the different departments were contacted in February 2012 by e-mail. Those willing to participate informed the nursing staff in their departments and then gave the contact details of the nurses who were willing to take part in the study. Additional participants were acquired using a snowball approach, particularly through well-connected interviewees who told their co-workers about the study and asked them to get in touch with the investigator if they were interested. Purposive sampling was employed in order to ensure that nurses were from a range of fields, hierarchical positions and work experience and that they were non-vaccinated and working in units with high-risk patients.

### Data collection

Interviews were conducted during spring and fall 2012. Interviews were conducted in Swiss or High German, depending on the participant’s preference; they lasted an average of 30 min and were audio-recorded. Additionally, field notes were made by the investigator shortly after the interviews. The interview partners were informed that their statements would be used in a thesis and possibly in a published research article. Oral consent was obtained from all interview partners and was documented at the beginning of each oral recording. All recordings were transcribed verbatim using High German diction. A.P., who is a native Swiss German speaker, conducted and transcribed the interviews and also translated the Swiss German interviews into High German. There was no external validation of the translations. Analysis was conducted using the High German transcription.

Interviews were typically conducted at participants place of work or a public venue. Only A.P. and the respective interviewee were present. A semi-structured interview tool about nurses’ reasons for declining influenza vaccination was applied to give a frame to the conversation and follow-up questions were asked based on the interviewees’ responses. After 18 interviews the question about data saturation arose and was discussed by the research team. It was agreed that concerning the main themes saturation was reached and that no new major discrepancies were coming up during the interviews. No repeat interviews were carried out and the transcripts were not returned to participants.

### Data analysis

Using the interview transcriptions, A.P. and S.M. performed conventional content analysis, [18] focusing on themes common across participants as well as those unique to individuals that may offer insight into differences in perspectives and discrepancies in practice. Initial themes discovered in the interviews were labelled using a process of open coding, codes emerged directly from the data. Two investigators [D.S., B.E.] reviewed the initial analysis to clarify and refine codes and A.P. and D.N. later reviewed the analysis. Coding differences were resolved via discussion.

## Results

### Characteristics of respondents

A total of 18 nurses were interviewed, 14 of which were female. Participants’ work experience ranged from 1–37 years (mean 14.4). Nurses worked in six different units with high-risk patients (haematology, cardiology, nephrology, geriatrics, ICU, oncology) and held various hierarchical positions. Seventeen nurses spent more than half of their working time with patients directly.

### Reasons for declining vaccination

**Fig. 1:**
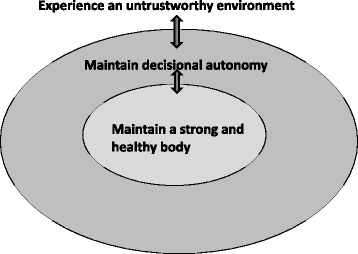
Nurses’ reasons for declining seasonal influenza vaccination

Reasons for declining influenza vaccination included a broad variety of themes that were structured into three recurring main themes. The first theme in the narratives was the strong emphasis of the interviewed nurses “maintaining a strong and healthy body”. The second theme described by nurses was the importance of “protecting decisional autonomy” in order to take care of themselves and others. The theme of “perception of an untrustworthy environment” illustrates the nurses’ perception of health authorities, pharmaceutical companies and scientists, which are often seen as opposing, non-trustworthy authorities. This view which seems to influence the nurses’ decision making process. These three main themes did not stand alone, but were connected: For most nurses decisional autonomy was crucial in order to maintain a strong and healthy body, which seemed to be the core theme. This was especially important due to the common perception of being surrounded by an untrustworthy environment, which affected their autonomy and therefore also seemingly posed a threat to maintaining a strong and healthy body. These interrelations and dependencies are depicted in Fig. 1.

### Maintaining a strong and healthy body

The interviewed nurses emphasized the desire to maintain a strong and healthy body. While some interviewees did not perceive influenza as a threat to their health and well being and therefore did not find a vaccination necessary, others felt the vaccine would not promote their health, due to lack of efficacy, or would even harm their health due to negative side-effects or a weakening effect on the immune system.

Nearly all of the nurses expressed the belief that influenza did not pose a threat for them since they were healthy, did not belong to the high-risk population and had never before fallen ill with influenza. They therefore found it unnecessary to get the vaccination. As one nurse explained: *“Well, I don't want to get vaccinated because I have not had it before, because I don’t find it reasonable, because I am a healthy person and not at risk myself if I don't get vaccinated.” N15*

Fear of side effects was also a commonly stated theme. Some nurses had felt ill after having been vaccinated against influenza in the past. This ranged from feeling sick for a couple of days to one nurse who recalled whole months of being unwell after she had been vaccinated: *“But my experience is that whenever I got the vaccination I felt bad for half a year. Felt bad for a couple of months.” N2* Participants appeared to ascribe these negative effects to the influenza vaccination and some reported that they based their decision not to get vaccinated on a negative experience they had more than a decade ago.

Ten of the nurses had never been vaccinated against influenza before and therefore had no personal experience. However, nearly all of the nurses had observed side effects in colleagues or family members or heard about such cases from hearsay. This seemed to be a very current topic and often discussed among colleagues:

*"And it is also true that you sometimes hear about those people who got the vaccination and nonetheless become ill, right, and that people also say: "Well, it could be that you have to stay in bed because of the vaccination." So, because of that…I don't get it." N10*

Others reported negative experiences with vaccination or medication in general, which had nothing to do with the influenza vaccine, but which led to their reluctance to get vaccinated:

*“I had a major reaction to a vaccination in my early years as a nurse. The whites of my eyes are still coloured a bit yellow from this…I was seriously ill. Three weeks with enlarged lymph nodes and furthermore I was afraid I had something serious!…But I`m not against vaccinations in general, I`m just against vaccinations for myself.” N1*

The belief that the vaccine would affect the immune system in a negative way by weakening it or that experiencing influenza was beneficial and a natural process was also mentioned several times. As one nurse stated:

“*I`m generally questioning whether it makes sense to manipulate the immune system in such a way and to vaccinate it with anything and everything, so that it can`t develop its own defences, right?” N2*

### Protecting decisional autonomy

Protecting their own autonomy was another important theme for the interviewed nurses; this argument included the right to bodily integrity, but also the right to fall ill and the right not to be pressured into doing something by superiors.

The right to bodily integrity and self-determination were cited universally by nurses as crucial issues. As one nurse explained: *“No, I`m the one in charge of my body. And nobody else…For me this is the only thing where I can consequently go through with something and therefore I am doing this with my body. No one is going to stick a needle into my body.” N7*

Furthermore, some nurses saw it as their right to become ill and stay at home, especially since they did so much for others at work. They did not want this perceived right to fall ill to be taken from them by their superiors: *“When that began I also had the idea…well that one should have the right to be ill, so to speak. That if there`s too much stress at work that you don`t have as much immune resistance…that you can stay at home for a week and don`t have to go to work by all means.” N6*

Others feared that accepting the influenza vaccine would entail more vaccinations or other measures restricting their autonomy:

*“Because there`s always something new, isn’t there? It won’t just stop with the influenza vaccination but then there will be something like with the swine…or something like that. You can’t dictate, the hospital can’t dictate "don`t go partying, young people. You’ll fall ill more easily if you go to a club." or something like that. There`s always people drawing bigger circles, right. Smoking, because the vocal cords dry out and then you will be more likely to get an infection. No obesity, then you will be more likely to miss work. There is a line somewhere, when you begin to affect personal rights.” N1*

Some nurses were of the opinion that the hospital or their superiors put them under too much pressure to be vaccinated. They did not want their decisional autonomy to be curtailed by pressure from their superiors and did not appreciate the moralizing tone and how the emphasis was put on the patients’ wellbeing and not their own:

*“The other thing that has always bothered me is those campaigns that have been made. That you're under pressure like that. That the nursing staff has to do it and that you basically have to have a bad conscience if you don't get vaccinated…because us bad nurses will infect the patients that way…something like that.” N6*

### Perception of an untrustworthy environment

A further theme, which was brought up by the interviewed nurses was a lack of trust; be it a lack of trust in the efficacy and safety of the influenza vaccine itself or a lack of trust in those individuals or health authorities promoting and selling the vaccine.

Some nurses argued that there was more than one virus and the viruses undergo mutations. Since the composition of the vaccine was only based on assumptions of what this season’s influenza virus would look like and the fact that the development and production of the vaccine had to happen very quickly every year, they thought the vaccine would unlikely be effective: *“Because the vaccination is only developed when influenza is already here and you can only get vaccinated against one virus. If that's the virus I would have infected myself with, or if I couldn't still fall ill from something else…” N6*

Another fear was that the vaccine was unsafe due to carrier substances. Since the vaccine needed to be produced quickly every year, some suspected sloppiness in the mode of production:

*“Yes, and I also don`t believe that it`s always pure and stuff because it's always done fast, under pressure. Everyone has to be the first, that`s the one who can put it on the market. And like with everything that happens in a hurry.. there`s no more regard for thoroughness, for cleanliness. When they`re under pressure like that, then it`s all about.. it`s about big fat bonuses, that`s what it`s also about.” N7*

Mistrust of the incentive of promoting influenza vaccine was also stated. Many of the nurses believed that economic interests were involved and that the concern for their, or the patient’s, health was only a charade. They expressed mistrust, mainly in pharmaceutical companies, but also in physicians and hospital management.

This mistrust was often enhanced by the experience of the Swine flu epidemic of 2009 – a topic, which arose in three-quarters of all interviews and generally had a negative effect on their perception of the influenza vaccine and the decision to receive it. Nurses believed the issue had been overhyped and had only lead to the pharmaceutical companies getting rich:

*Well I got the vaccination every year…until last year. And this year there was this hype with this…this hype because of this H1N1 where they told us here in this unit.. I don't think it was just this unit even, but…basically they forced us: "If you don't do this, then it's your fault, basically, if a patient dies." That's put in a provocative way…And, like they finally found out, nothing really happened. To the contrary, the pharma companies got rich…that's why this year, or last season I said: "No, I'm not doing this anymore." N8*

Indeed, some saw vaccination as a big scam and even suspected a conspiracy between physicians and pharmaceutical companies. *“From my point of view all it's really about is the money. It's not about the patient… I think there's a Mafia between the doctors and the pharma industry. They both benefit from each other. It's a "lucrative" deal, it has to be produced quickly and the pharma industry makes a lot of money from it.” N7*

Other nurses doubted the authenticity of studies showing the efficacy of influenza vaccine:

*“But I think these numbers, they…I see this with other studies because I don't believe a thing anymore. In all these years "oh what has been proven…" and the next day there's a new doctor and a new thing, a new study and then everything is just cold coffee again, to put it simply but that's how I experienced it in all these years and that's why… Like I said, I don't believe anything anymore, that somebody just tells me.” N7*

All this led to doubts concerning the true motivations and credibility of those promoting the influenza vaccination.

In order to overcome mistrust and make an autonomous decision nurses reported desiring information to be provided to them in a more personal and interactive manner: *“If there was further education.. further education classes, which you had go to, which were mandatory, right? If this were to be made transparent…if studies would be published on how many patients actually get infected by the staff…and the numbers are impressive, then I think that would have a signalling effect.” N5*

Overall the perception of an untrustworthy environment appeared more pronounced the less knowledgeable nurses were in respect to seasonal influenza prevention and the less problem awareness they had. Many reported that information about the issue was lacking; they had not been thoroughly informed by their superiors and had never seen the recommendations that HCWs should receive the annual influenza vaccination: *“Like I said, at the moment I'm just like…not informed. I didn't even know about the 70-90% protection....I somehow have no idea about it and well…why should I get it?” N13*

Furthermore, there was often little awareness among nurses about why influenza vaccination might be important, for example, to protect vulnerable patients. Nurses had not thought about the matter or were simply not interested in the question: *“For one thing, it's also to a certain degree of laziness…to concern myself with this.” N14*

## Discussion

Three interconnected themes explaining why nurses decline influenza vaccination were identified: Firstly, the idea of maintaining a strong and healthy body, which was a central motif for rejecting the vaccine. Secondly, the wish to maintain decisional autonomy - especially over one’s body and health. Thirdly, nurses’ perception of being surrounded by an untrustworthy environment, which restricts their autonomy and seemingly is in opposition to their goal of maintaining a strong and healthy body.

Maintaining a strong and healthy body is a main theme for nurses rejecting influenza vaccination; interestingly, the vaccine is not perceived as beneficial but in contrast even seen as a potential danger to their health. Many of the interviewed nurses for example believed people regularly became sick because of vaccination, contrary to the evidence on this issue [19]. In line with other research, seeing no personal risk, fears about side effects, and the perception that the vaccine is not effective were also key reasons for nurses not to get vaccinated [10, 11, 13]. This might show a stronger reliance on concrete experiences or public beliefs such as for instance the often negative reports on influenza vaccination and vaccination in general by the media [20, 21] compared to the more abstract research evidence. Importantly our study shows that in the line of these beliefs, the preservation of a healthy body is at the forefront for the nurses. Caring for oneself in order to be able to work professionally is something that has been integrated in nurses’ training in the last decades. In contrast, Swiss nursing education at the beginning of the twentieth century strongly focused on the ideal of a self-sacrificing, caring approach that led to existential exhaustion for many nurses [22].

In our study, in order to protect their personal needs, many interviewees called upon their autonomy – their right to choose and to maintain physical integrity. In contrast, a number of commentators have come to the conclusion that there is a moral duty for HCWs to get the influenza vaccination when evaluating the rivalling ethical values of personal autonomy and patient protection [23, 24]. Consistent with previous research showing that HCWs who get vaccinated do so primarily to protect themselves, then their family, friends and colleagues, whereas patient safety only takes third place [13, 25] the protection of patients was no or only a minor issue in the narratives of our participants. Moreover, a discourse on the rivalling ethical values was non-existent. The strong emphasis on being autonomous from determining authorities, in order to maintain a strong and healthy body may be connected to the professional emancipation over decades. It might be argued that these attitudes and values have been shaped to some extent by the development of the nursing profession from a self- sacrificing profession dominated by others to a self-determined profession with nurses defining the standards of their work. Many of the interviewed nurses thought that too much was being asked from them in general, they were unwilling to “give more”, particularly since they did not receive much recognition in return. It is therefore not surprising that moralizing pressure by authorities, in the context of non- mandatory influenza vaccination recommendations, led to more emphasis on autonomy and - in order to keep autonomy - rejection of vaccination.

The perception of being surrounded by an untrustworthy environment seems to add to this rejection. This mistrust possibly stems from the discrepancy between the claim for self-determination and for demarcation from traditional hierarchies on one hand and a lack of academic education concerning evidence based decision-making and thus a certain dependency on aforementioned authorities on the other hand. This might be one of the reasons to explain the differences in intervention effects in nurses and physicians [7, 8]. The “one size fits all” approach of educational intervention components might not work. Although nurses worked in institutions where evidenced based medicine is advocated and practiced they were insecure to trust studies and science. As Sampson and colleagues have stated, “[a]necdotal evidence is often more powerful than established evidence” [26]. In Switzerland for example efforts have been made on a national level to provide information on the vaccine, still lack of knowledge and awareness of the issue were often criticized by nurses. The impression arises that the Swiss Federal Office of Public Health’s (Bundesamt für Gesundheit - BAG) influenza prevention campaign with brochures, flyers and posters are not having the desired effect on this target group [27].

There seems to be a lack of professional information seeking and processing, ethical reasoning and decision-making competence. Possibly, it is not (only) information, which is lacking but better tools to facilitate the decision-making process and to gain a better understanding of the benefit and risks of the vaccination and come to an evidence-based decision. There is a much larger variety of educational backgrounds in nurses than other professions. In Switzerland the teaching curricula changed significantly during the last ten years and the academic nursing education was only initiated a decade ago. Therefore clinical reasoning – decision-making based on research evidence - has only recently been integrated in nurses’ education. This educational situation is comparable with other European countries given only a few exceptions.

Participating hospitals were situated in just two cantons of Switzerland. However, the percentage of nurses who come from adjacent European countries is known to be considerable in the two cantons. We have therefore reason to believe that our sample represents a variety of views that go beyond a typical “Swiss German” culture. The small sample may limit the generalisability of our findings, but data saturation – which indicates an adequate sample in qualitative studies - was reached. As is in all interview studies, research of this nature relies on consenting participants, increasing the chance of a biased sample; nurses who came forward were possibly those with a more pronounced opinion on this topic. However, as the aim was to identify barriers, this possible bias does not invalidate results. In addition, given the widespread presence of the three identified themes, it is likely that we have identified real and widely prevalent concerns that decrease uptake of influenza vaccination.

## Conclusion

In conclusion, the wish to maintain a strong and healthy body seems to stand at the core for many nurses. Their autonomy plays an important role in protecting their body against an environment, which is often perceived as untrustworthy. This mistrust stems to some extent from a discrepancy between the claim for self-determination and a dependency on traditional authorities. Nurses tend to rely on conventional health beliefs rather than evidence based medicine when making their decision to decline influenza vaccination. It is important to identify and acknowledge these interrelations. It seems that in order to reach nurses, interventions to increase influenza vaccination should be tailored specifically for this group instead of applying a “one size fits all” approach. As pressure from above leads to further rejection of the vaccine and information alone has been shown to have little effect in this group, empowering nurses by promoting decision making skills and by strengthening their appraisal may be important factors to consider when planning future interventions to improve vaccination rates. The teaching of evidence based decision-making should be integrated on different levels, including nurses’ training curricula, their workspace and further education. In order to strengthen the profession and to promote credibility and trust there is a need for self-empowerment among nursing professionals to active leadership.

## Abbreviations

HCWs: Healthcare workers

## References

[1] Hayward AC, Harling R, Wetten S, Johnson AM, Munro S, Smedley J (2006). Effectiveness of an influenza vaccine programme for care home staff to prevent death, morbidity, and health service use among residents: cluster randomised controlled trial. BMJ.

[2] Ajenjo MC, Woeltje KF, Babcock HM, Gemeinhart N, Jones M, Fraser VJ (2010). Influenza vaccination among healthcare workers: ten-year experience of a large healthcare organization. Infect Control Hosp Epidemiol.

[3] Mereckiene J, Cotter S, Nicoll A, Lopalco P, Noori T, Weber J (2014). Seasonal influenza immunisation in Europe. Overview of recommendations and vaccination coverage for three seasons: pre-pandemic (2008/09), pandemic (2009/10) and post-pandemic (2010/11). Euro Surveill.

[4] Zhang J, While AE, Norman IJ (2012). Seasonal influenza vaccination knowledge, risk perception, health beliefs and vaccination behaviours of nurses. Epidemiol Infect.

[5] Christini AB, Shutt KA, Byers KE (2007). Influenza vaccination rates and motivators among healthcare worker groups. Infect Control Hosp Epidemiol.

[6] O'Lorcain P, Cotter S, Hickey L, O'Flanagan D, Corcoran B, O'Meara M (2014). Seasonal influenza vaccine uptake in HSE-funded hospitals and nursing homes during the 2011/2012 influenza season. Ir Med J.

[7] Friedl A, Aegerter C, Saner E, Meier D, Beer JH (2012). An intensive 5-year-long influenza vaccination campaign is effective among doctors but not nurses. Infection.

[8] Tapiainen T, Bar G, Schaad UB, Heininger U (2005). Influenza vaccination among healthcare workers in a university children's hospital. Infect Control Hosp Epidemiol.

[9] Balmer D, Plattner T, Haederli A. Umfrage zur Grippeimpfung. Clinical Infectious Diseases. 2015;50:459–64. Retrieved from http://dok.sonntagszeitung.ch/2015/spitalgrippe/. Accessed 25 Apr 2017.

[10] Rhudy LM, Tucker SJ, Ofstead CL, Poland GA (2010). Personal choice or evidence-based nursing intervention: nurses' decision-making about influenza vaccination. Worldviews Evid Based Nurs.

[11] Hollmeyer HG, Hayden F, Poland G, Buchholz U (2009). Influenza vaccination of health care workers in hospitals--a review of studies on attitudes and predictors. Vaccine.

[12] Shahrabani S, Benzion U, Yom Din G (2009). Factors affecting nurses' decision to get the flu vaccine. Eur J Health Econ.

[13] Prematunge C, Corace K, McCarthy A, Nair RC, Roth V, Suh KN (2014). Qualitative motivators and barriers to pandemic vs. seasonal influenza vaccination among healthcare workers: a content analysis. Vaccine.

[14] Zhang J, While AE, Norman IJ (2011). Nurses' knowledge and risk perception towards seasonal influenza and vaccination and their vaccination behaviours: a cross-sectional survey. Int J Nurs Stud.

[15] Lehmann BA, Ruiter RA, van Dam D, Wicker S, Kok G (2015). Sociocognitive predictors of the intention of healthcare workers to receive the influenza vaccine in Belgian, Dutch and German hospital settings. J Hosp Infect.

[16] Willis BC, Wortley P (2007). Nurses' attitudes and beliefs about influenza and the influenza vaccine: a summary of focus groups in Alabama and Michigan. Am J Infect Control.

[17] Richards D, Rahm Hallberg I. Complex Interventions in Health: An overview of research methods: Routledge, New York; 2015.

[18] Hsieh HF, Shannon SE (2005). Three approaches to qualitative content analysis. Qual Health Res.

[19] Rue-Cover A, Iskander J, Lyn S, Burwen DR, Gargiullo P, Shadomy S (2009). Death and serious illness following influenza vaccination: a multidisciplinary investigation. Pharmacoepidemiol Drug Saf.

[20] Lehmann BA, Ruiter RA, Kok G (2013). A qualitative study of the coverage of influenza vaccination on Dutch news sites and social media websites. BMC Public Health.

[21] Neue Zürcher Zeitung. Swissmedic stoppt Auslieferung von Novartis-Grippeimpfstoff. Neue Zürcher Zeitung 2012. Retrieved from: http://www.nzz.ch/aktuell/schweiz/swissmedic-stoppt-auslieferung-vonnovartis-grippeimpfstoff-1.17715608. Accessed 15 July 2013.

[22] Fritschi A (1990). Schwesterntum: Zur Sozialgeschichte der weiblichen Berufskrankenpflege in der Schweiz1850-1930.

[23] McLennan S, Gillett G, Celi LA (2008). Healer, heal thyself: health care workers and the influenza vaccination. Am J Infect Control.

[24] van Delden JJ, Ashcroft R, Dawson A, Marckmann G, Upshur R, Verweij MF (2008). The ethics of mandatory vaccination against influenza for health care workers. Vaccine.

[25] Wicker S, Doerr HW, Gottschalk R, Rabenau HF, Allwinn R (2007). Influenza: acceptance of vaccination by healthcare personnel. Evaluation of the influenza season 2006/2007. Dtsch Med Wochenschr.

[26] Sampson R, Wong L, Macvicar R (2011). Parental reasons for non-uptake of influenza vaccination in young at-risk groups: a qualitative study. Br J Gen Pract.

[27] Gesundheit Bf. Grippe? Impfen macht Sinn Eine Information für Fachpersonen im Gesundheitswesen. Bern; 2008.

